# Knowledge, attitude, practice, and perception among healthcare workers on COVID-19 and acceptance of available vaccines at Shree Hindu Mandal Hospital, Dar es Salaam, Tanzania

**DOI:** 10.11604/pamj.2024.49.111.39563

**Published:** 2024-12-09

**Authors:** Mohamed Zahir Alimohammed, Raidah Gangji, Shahista Jaffer, Gibson Kagaruki, Anna Jazza, David Andimile, Kaushik Ramaiya

**Affiliations:** 1 Research and Training Department, Shree Hindu Mandal Hospital, P. O. Box 581, Dar es Salaam, Tanzania,; 2 Department of Haematology and Blood Transfusion, Muhimbili University of Health and Allied Sciences, P.O.Box 65001, Dar es Salaam, Tanzania,; 3 Department of Biochemistry and Molecular Biology, Muhimbili University of Health and Allied Sciences, P.O.Box 65001, Dar es Salaam, Tanzania,; 4 Tanzania Human Genetics Organization, Dar es Salaam, Tanzania,; 5 Department of Genetics, University Medical Center Groningen, University of Groningen, Groningen, the Netherlands,; 6 Huberk Kairuki Memorial University, P.O.Box 65300, Dar es Salaam, Tanzania,; 7 National Institute for Medical Research, Tukuyu Research Center, P.O.Box 538 Tukuyu, Tanzania

**Keywords:** Health care workers, knowledge, attitude, vaccine uptake, COVID-19, Tanzania

## To the editors of the Pan African Medical Journal

The COVID-19 pandemic has placed unprecedented pressure on healthcare workers (HCWs) globally, with African settings facing unique constraints due to limited healthcare resources and infrastructure. Healthcare workers, who include doctors, nurses, and various support staff, are at increased risk for exposure to SARS-CoV-2, underscoring the importance of widespread vaccination to protect frontline workers. However, the success of such vaccination programs depends largely on understanding and addressing factors that influence HCWs´ knowledge, attitudes, practices, and perceptions (KAPP) about COVID-19 and vaccines. Identifying these factors can guide interventions and prepare HCWs to lead public health initiatives effectively [1,2].

In this study, conducted at Shree Hindu Mandal Hospital (SHMH) in Dar es Salaam, Tanzania, we aimed to assess HCWs' KAPP toward COVID-19 and evaluate their COVID-19 vaccine acceptance. This cross-sectional survey, conducted between March and June 2021, included 150 consenting HCWs from an eligible population of 299 employees at SHMH, a private tertiary hospital (Table 1). Participants represented a diverse group, including medical doctors, nurses, pharmacists, laboratory technicians, and non-medical staff members. A structured questionnaire captured demographic information and assessed COVID-19 knowledge, attitudes, practices, perceptions, and vaccine acceptance. Each KAPP element was evaluated using Bloom´s criteria, with scores categorized as low, moderate, or high [3].

**Table 1:** demographic characteristics of the healthcare workers respondents employed at Shree Hindu Mandal Hospital

Variable	Categories	Frequency n (%)
**Sex**	Male	73 (42.9)
	Female	97 (57.1)
**Ages**	Mean (SD)	33.1 (9.9)
	<30 years	82 (48.2)
	30-49 years	79 (46.5)
	>50 years	9 (5.3)
**Marital status**	Not married	94 (55.3)
	Married	76 (44.7)
**Profession**	Accountant	10 (5.9)
	Lab technicians	7 (4.1)
	Medical doctor	36 (21.2)
	Nurse	90 (52.9)
	Others	4 (2.4)
	Pharmacists	17 (10.0)
	Radiographer	6 (3.5)
**Experience of healthcare workers with COVID-19**	Have you recently been infected with COVID-19? (YES)	22 (12.9)
	Have you previously been infected with COVID-19? (YES)	65 (38.2)
	Have you ever had a family member or friend infected with COVID-19? (YES)	89 (52.3)
	Do you feel a stigma is associated with any individual affected by COVID-19? (YES)	84 (49.4)
	Have any of your family members or friends died due to COVID-19? (YES)	73 (42.9)
**Main source of COVID-19 pandemic information?**	World Health Organization (WHO)	34 (20.0)
	National Centre of Disease Control (NCDC)	8 (4.7)
	News and social media	43 (25.3)
	Medical journals (internet)	17 (10.0)
	More than one source	65 (38.2)
	Other	3 (1.8)

Results indicate that only 21.8% of HCWs demonstrated high knowledge of COVID-19. Medical doctors and pharmacists exhibited the highest knowledge levels; however, the overall knowledge among other HCWs was moderate to low. This gap could be attributed to varying access to training and diverse information sources. For instance, many HCWs reported relying on social media and other mass media sources, which, while accessible, often spread unverified information. This reliance may affect the HCWs´ accurate understanding of COVID-19, potentially impacting their practice and decision-making regarding preventive measures [4,5].

Only 10% of participants expressed a positive perception of COVID-19, revealing a limited recognition of the virus's severity and transmissibility. These results suggest a need for more consistent and accurate health communication, especially considering positive perception is often associated with better adherence to preventive practices. The low perception scores might also reflect HCWs´ concern over stigmatization or the additional stress COVID-19 has placed on healthcare systems, particularly in low-resource settings like Tanzania [6,7].

Despite the knowledge and perception gaps, COVID-19 vaccine acceptance was relatively high, with 69% of HCWs indicating they would accept the vaccine. An impressive 74.7% reported that they would encourage family members and patients to get vaccinated. Higher acceptance was noted among doctors and pharmacists, who often have better access to scientific information and are more likely to be familiar with clinical research. Our findings align with studies across sub-Saharan Africa where HCWs in clinical roles show greater vaccine acceptance due to their scientific literacy and exposure to infectious disease management [8,9].

Positive attitudes and good practices towards COVID-19 prevention were observed in 48.8% of the HCWs. These practices included consistent mask-wearing, regular hand hygiene, and social distancing. Younger HCWs and those without a recent COVID-19 infection history were more likely to engage in these preventive measures. This disparity suggests that non-medical HCWs may lack adequate training on infection control, highlighting the importance of extending structured training and resources to all categories of HCWs, especially those less directly involved in clinical care [10]. These findings have several implications for future public health initiatives. Strengthening reliable COVID-19 information channels for HCWs is crucial for building a well-informed workforce capable of promoting health measures effectively. Educational programs should incorporate clear, consistent messaging on COVID-19 risks and preventive practices. Such training should extend beyond clinical staff to include non-medical HCWs, who play an essential role in maintaining a safe healthcare environment. Additionally, campaigns to address and reduce the social stigma associated with COVID-19 can improve HCWs' willingness to discuss the disease openly, further enhancing public trust [8].

Furthermore, addressing misconceptions and enhancing HCWs' understanding of the risks of infectious diseases is vital to strengthening their role as public health advocates. Awareness campaigns that emphasize the preventive nature of COVID-19 measures and the role of vaccines in reducing severe outcomes could positively impact attitudes, aligning HCWs´ views more closely with their practice and influence in the community. Such campaigns will not only foster vaccine acceptance but also empower HCWs to lead by example, reinforcing public health directives and encouraging widespread adherence to health guidelines.

## Conclusion

Our study reveals significant gaps in HCWs' knowledge, perception, and practices related to COVID-19, which could hinder effective public health responses in Tanzania. Addressing these gaps through targeted information dissemination, structured training, and anti-stigma campaigns could bolster vaccine acceptance and enhance HCWs´ role in supporting immunization efforts. By equipping HCWs with reliable information and resources, healthcare institutions can create a more resilient frontline workforce prepared to respond to current and future health crises.
